# Spectroscopic Investigations of Porphyrin-TiO_2_ Nanoparticles Complexes

**DOI:** 10.3390/molecules28010318

**Published:** 2022-12-30

**Authors:** Andra Dinache, Simona Nistorescu, Tatiana Tozar, Adriana Smarandache, Mihai Boni, Petronela Prepelita, Angela Staicu

**Affiliations:** 1 National Institute of Laser, Plasma and Radiation Physics, 409 Atomistilor Str., 077125 Magurele, Romania; 2 Department of Biochemistry and Molecular Biology, Faculty of Biology, University of Bucharest, 91–95 Splaiul Independentei, 050095 Bucharest, Romania; 3 ELI-NP, “Horia Hulubei” National Institute for Physics and Nuclear Engineering, 30 Reactorului Str., 077125 Magurele, Romania

**Keywords:** PDT, porphyrin, titanium dioxide nanoparticles, spectral analysis, singlet oxygen quantum yield

## Abstract

This study presents the spectral characterization of TiO_2_ nanoparticles (NPs) functionalized with three porphyrin derivatives: 5,10,15,20-(Tetra-4-aminophenyl) porphyrin (TAPP), 5,10,15,20-(Tetra-4-methoxyphenyl) porphyrin (TMPP), and 5,10,15,20-(Tetra-4-carboxyphenyl) porphyrin (TCPP). UV-Vis absorption and Fourier transform infrared spectroscopy–attenuated total reflection (FTIR-ATR) spectroscopic studies of these porphyrins and their complexes with TiO_2_ NPs were performed. In addition, the efficiency of singlet oxygen generation, the key species in photodynamic therapy, was investigated. UV-Vis absorption spectra of the NPs complexes showed the characteristic bands of porphyrins. These allowed us to determine the loaded porphyrins on TiO_2_ NPs functionalized with porphyrins. FTIR-ATR revealed the formation of porphyrin-TiO_2_ complexes, suggesting that porphyrin adsorption on TiO_2_ may involve the pyrroles in the porphyrin ring, or the radicals of the porphyrin derivative. The quantum yield for singlet oxygen generation by the studied porphyrin complexes with TiO_2_ was higher compared to bare porphyrins for TAPP and TMPP, while for the TCPP-TiO_2_ NPs complex, a decrease was observed, but still maintained a good efficiency. The TiO_2_ NPs conjugates can be promising candidates to be tested in photodynamic therapy in vitro assays.

## 1. Introduction

In the last years, photodynamic therapy (PDT) has attracted significant interest for cancer treatment and the inactivation of microorganisms [1]. PDT implies a combination of photophysical and photochemical processes that lead to biological effects. Photosensitizers (PS) are well known for their use in PDT [2] or in Antimicrobial Photodynamic Therapy (APDT) [3]. Visible light irradiated PS have high efficiency in generating very reactive species such as singlet oxygen, which can destroy the cancer cell or kill various pathogens [4].

In subsequent photon absorption, the PS molecules are excited to their singlet state S_1_. Part of the energy is emitted as fluorescence and/or is lost in internal conversion by decaying back on the ground energy state, and the remaining energy directs PS molecules to the excited triplet state [5,6]. PSs can follow two types of mechanisms of photodynamic reaction, both being closely dependent on oxygen molecules inside cells.

For Type I, in the excited triplet state, the PS can transfer energy to the biomolecules from its surroundings. Between the PS in the triplet state and the disease tissue (or substrate), a hydrogen or electron is transferred, which leads to the formation of free radicals and anion radicals of the PS and the substrate. Electrons interact with oxygen molecules, which remain in their ground energetic state. This process leads to the production of reactive oxygen species (ROS), initially in the form of superoxide anion radical (•O_2_^−^), which creates further generation of ROS inside the cells. The initiated cascade of reactions leads to oxidative stress, resulting in the destruction of cancer or pathogen cells [7].

Alternatively, in a type II PDT mechanism, as a result of the photosensitizer’s transition into the excited triplet state, energy is transferred directly to the oxygen molecule in the ground state (a triplet state). Direct energy transfer between molecules (PS → O_2_) is possible because they have the same spins. In this way, excited oxygen particles called singlet oxygen (^1^O_2_) are generated, which are characterized by extremely strong oxidizing properties [7]. Both mechanisms can induce tumoral cell or pathogen deaths [8].

APDT is a promising alternative treatment for infections that are resistant to antibiotics [9,10,11].

Significant attention among the many PS that are studied in biological experiments is given to porphyrins, a class of aromatic macrocycles made up of four pyrrole units linked by methine bridges. Porphyrin derivatives are dyes of synthetic or natural origin [12], which play essential roles due to their functional flexibility in biosystems and PDT [13,14,15]. The aromatic characteristic of porphyrins enables excellent visible light absorption attributed to π–π* electronic transitions. Substitutions at the α, β, γ, and δ positions with different functional groups can customize the photophysical properties of these materials, and their subsequent applications [16]. Porphyrins have a series of remarkable physical and chemical properties such as photostability, phototoxicity, and singlet oxygen production by irradiation with visible light. Furthermore, these macromolecules are considered benchmarks in biological sciences as potential photosensitizing agents in anticancer, antiviral, antifungal, antibacterial, and antiparasitic PDT [17,18].

PDT is increasingly being used to treat cancer and bacterial infections, but it is also being used to treat a variety of other diseases, such as rheumatoid arthritis, atherosclerosis, and macular degeneration [19]. The biomedical application of PDT is also being explored for the cure of cardiovascular issues, ophthalmology, dermatology, urology, and infectious diseases [12,20,21].

In recent years, the active development of PDT has included the research for new PS and carriers for their delivery. Among the numerous potential approaches to photodynamic research, those that combined PS and nanoparticles (NPs) resulted in an increase in the PS selectivity and/or therapeutic efficacy [22,23,24]. NPs are a type of particle that range in size from 1 to 100 nm and include lipid micelles, liposomes, polymeric NPs, quantum dots, and metallic NPs [25,26]. The latter should be given special attention, and NPs such as Fe_2_O_3_, Au, ZnO, Ag, and TiO_2_ are an important class of NPs that showed an efficiency increase of many existing treatments in several studies [27,28]. Due to their high stability, controllable size, and optical characteristics, as well as their ease of surface functionalization to make them more biocompatible in biological applications, inorganic NPs have significant benefits over organic nanoparticles [29,30]. Drugs that are linked to NPs can improve their selective accumulation in tissues as well as their ability to penetrate cell membranes [23,27]. Titanium dioxide NPs, due to their capability to produce ROS and thus induce cell death, can be applied as photosensitizing agents [24]. The main drawback of TiO_2_ NPs is their light absorption in the UV spectral range [24]. Despite the material’s promising characteristics for PDT, many concerns related to tissue warming under the impact of light, low tissue penetration by ultra-violet light, and the damaging effect of UV radiation on the human body limit the use of TiO_2_ NPs in PDT. Therefore, the NPs surface is altered to enhance the ROS generation and to improve the physicochemical characteristics, including the absorption of visible light. For this purpose, porphyrins and phthalocyanines were most the frequently used photosensitizers. Porphyrins are suggested to shift the photocatalytic response of TiO_2_ to the visible region and prevent the electron–hole recombination [31,32]. In particular, 5,10,15,20-tetrakis(4-carboxyphenyl)-porphyrin and its zinc(II) derivative (ZnTCPP) enhances the quantum efficiency of TiO_2_ [33,34]. The oxidation of organic molecules occurs as a result of the photoinduced electron injection into TiO_2_’s conduction band and subsequent formation of ˙O_2_^−^ and ˙OH with O_2_ adsorbed on the surface of TiO_2_ [31]. Efficient electron transfer from the excited PS to the TiO_2_ conduction band requires good electronic coupling between the lowest unoccupied orbital (LUMO) of the PS and the Ti 3d orbitals. Effective coupling has typically been accomplished with carboxylate or phosphonate groups, which bind tightly to the TiO_2_ surface [33].

The PS-TiO_2_ complexes that were synthesized via either chemical functionalization or mixed covalent/noncovalent approach in organic solvents and in water [35] showed potential as catalysts for biomedical and environmental applications, as material for dye-sensitized solar cells, and as photosensitizers for PDT [24,36,37].

In the current study, the morphological and spectral characterization of TiO_2_ NPs conjugates with three porphyrin derivatives: 5,10,15,20-(Tetra-4-aminophenyl) porphyrin (TAPP), 5,10,15,20-(Tetra-4-methoxyphenyl) porphyrin (TMPP), and 5,10,15,20-(Tetra-4-carboxyphenyl) porphyrin (TCPP). The porphyrin loading efficiency on TiO_2_ NPs was analyzed by UV-Vis absorption spectroscopy. The adsorption of porphyrins on TiO_2_ surface was evidenced by FTIR-ATR spectroscopy, as well as scanning electron microscopy (SEM) and dynamic light scattering (DLS) measurements. The PS-TiO_2_ NPs efficiency in singlet oxygen generation was investigated via phosphorescence emission at 1270 nm.

## 2. Results and Discussions

### 2.1. Morphological Characterization of the Porphyrin-TiO_2_ Complexes

**Figure 1:**
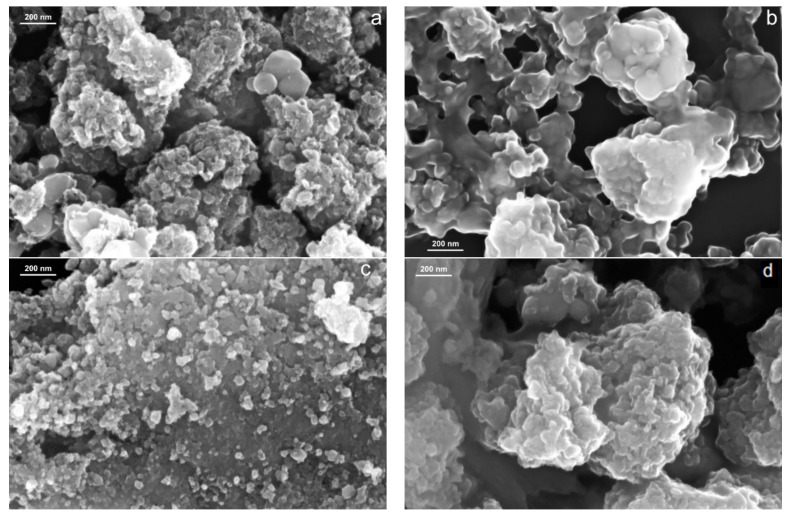
SEM images at 100,000× magnification of (**a**) TiO_2_ sample, (**b**) TiO_2_-TAPP sample, (**c**) TiO_2_-TCPP sample, (**d**) TiO_2_-TMPP sample.

The SEM images of TiO_2_ sample and the complexes of TiO_2_ with porphyrins (TAPP, TCPP, TMPP) are depicted in Figure 1a–d.

The irregular sphere-shaped particles have distinct structures, which can be observed from the SEM investigations (Figure 1a). The TiO_2_-porphyrin samples (see Figure 1b–d) have microspheres similar to the TiO_2_ sample (Figure 1a). In SEM analysis on TiO_2_-TCPP (Figure 1c) and TiO_2_ -TMPP (Figure 1d), it is obvious that the particles are dispersed on a nanometer scale.

In Figure 1a, the TiO_2_ sample shows a surface with nanoparticles with dimensions between 17.1–23.2 nm (Table 1), but for the TiO_2_-TAPP sample (Figure 1b), the size distribution is between 40.4–46.6 nm due to the aggregates formed by TAPP and TiO_2_ nanoparticles. The TiO_2_-TCPP and TiO_2_-TMPP samples have dimensions of 18.7–24.4 nm and 29.4–33.7 nm, respectively.

The results of EDS investigations for TiO_2_ and TiO_2_-porphyrin samples, mass percentage, and atomic percentage are presented in Table 1. Regarding the chemical composition of the TiO_2_ complexes, analysis showed mostly Ti content, O, C, and N atoms, without other elements. The structural and morphological results revealed that porphyrins were uniformly dispersed on the surface of the TiO_2_ and that the synthesis process has an important role in the quality of the TiO_2_-porphyrin complexes.

The mean hydrodynamic size and zeta potential of TiO_2_ and the porphyrin-NPs suspensions were measured by DLS. These results are shown in Table 2.

The TiO_2_ NPs mean hydrodynamic size was 559.1 nm, which shows that aggregates are present in the measured samples. The values obtained for porphyrin-NPs complexes are similar to each other: 1213.7 nm for TiO_2_-TAPP, 1148.4 nm for TiO_2_-TMPP, and 1225.8 nm for TiO_2_-TCPP; this indicates the presence of larger aggregates in suspensions and, at the same time, shows the formation of complexes. The larger values of the sizes could be explained firstly because DLS approximates the aggregates to a theoretical sphere. The size of this sphere is also influenced by the thickness of the solvation shell (electrical double layer) [38]. Secondly, the coalescence and compactization phenomena take place due to centrifugation forces during preparation, and these processes cannot be completely reversed when they were dispersed by ultrasonication [39]. The polydispersity indexes shown in Table 2 indicated a broad size distribution in the samples [40].

The zeta potential of TiO_2_ suspensions was −50.3 mV. The formed complexes have zeta potential values in the same range, as follows: TiO_2_-TAPP zeta potential was −46.8 mV, −44.1 mV for TiO_2_-TMPP, and −44.0 mV for TiO_2_-TCPP. The obtained values indicate a fairly good stability. In general, suspensions having zeta potential higher than −30 mV (in absolute value) can be taken into consideration for pharmaceutical applications [41].

### 2.2. UV Vis Absorption Spectroscopy

**Figure 2:**
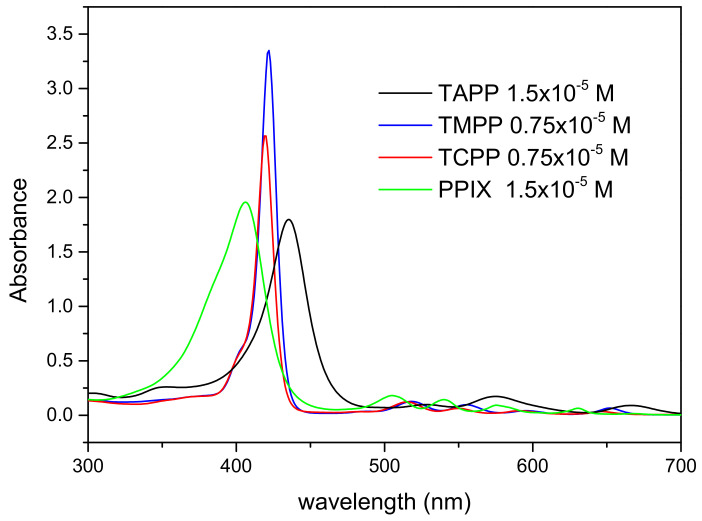
The absorption spectra of TAPP, TMPP, TCPP, and PPIX porphyrin solutions in DMF.

The absorption spectra of TAPP, TMPP, TCPP, and Protoporphyrin IX (PPIX) solutions in N-N-dimethylformamide (DMF) are shown in Figure 2. All compounds exhibit a Soret band between 400–450 nm as the main absorption feature, which is specific for porphyrins. The maxima of these are placed at 407 nm-PPIX, 419 nm-TCPP, 422 nm-TMPP, and 435 nm-TAPP. All porphyrins also show lower intensity Q absorption bands between 480–700 nm.

**Figure 3:**
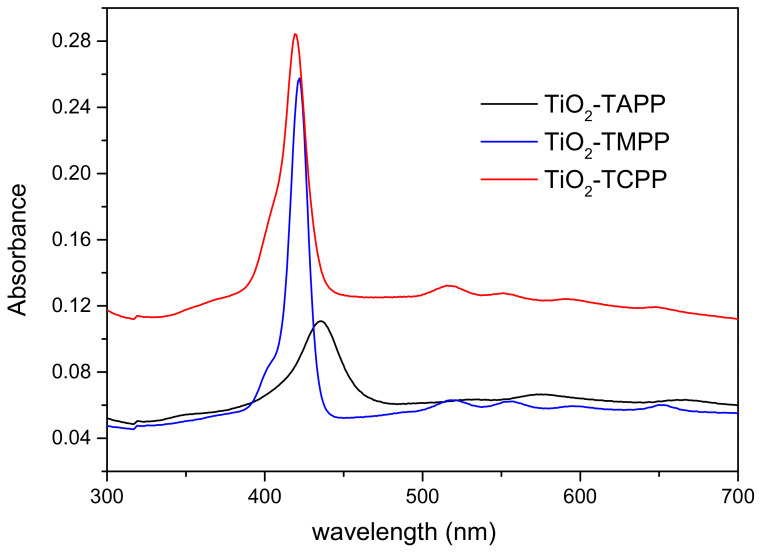
The absorption spectra of porphyrin-functionalized TiO_2_ nanoparticle suspensions.

The absorption spectra of porphyrin-functionalized TiO_2_ nanoparticle suspensions in DMF are shown in Figure 3. The characteristic absorption feature of porphyrins, the Soret bands arising at 400–450 nm, as well as lower intensity bands in longer wavelength spectral ranges can be observed. The nanoparticle loading with porphyrins can be determined by evaluating the absorbance of the major peak of the compounds, which is further compared with spectra obtained at known concentrations of the solutions (Figure 2). Thus, for all suspensions of TiO_2_ nanoparticles with a concentration of 0.024 mg/mL of TiO_2_, a concentration of porphyrins of 0.4 × 10^−6^ M is obtained.

The origin of the UV-Vis spectroscopic feature of TAPP was studied by Giovanelli et al. [42]. They found that several features are clearly due to the amino group of TAPP, whose effect on the UV-Vis spectrum consists of an overall red-shift and remarkable intensity changes within the Q band.

Electron-donor amino functionalization was found to reduce the HOMO–LUMO electronic transport gap and to increase the HOMO to HOMO−1 energy separation in a way that is consistent with an orbital destabilization process [42].

### 2.3. Fourier Transform Infrared Spectroscopy–Attenuated Total Reflection (FTIR-ATR)

The FTIR-ATR spectra of the porphyrin derivatives (TAPP, TMPP, TCPP) of TiO_2_ NPs and of TiO_2_ NPs loaded with each porphyrin were recorded. The frequencies observed for each porphyrin were compared with the theoretical frequencies, calculated with Gaussian 09 suite [43]. The assignation of the vibrations was conducted with Gauss View 5.0, which shows exactly the chemical bonds that vibrate for each calculated frequency, and was compared with specialized literature [43,44,45,46].

The observed frequencies for TAPP, as well as the ones calculated with Gaussian 09 suite, and the vibrations of the bonds assigned for each frequency, are given in Table 3.

**Figure 4:**
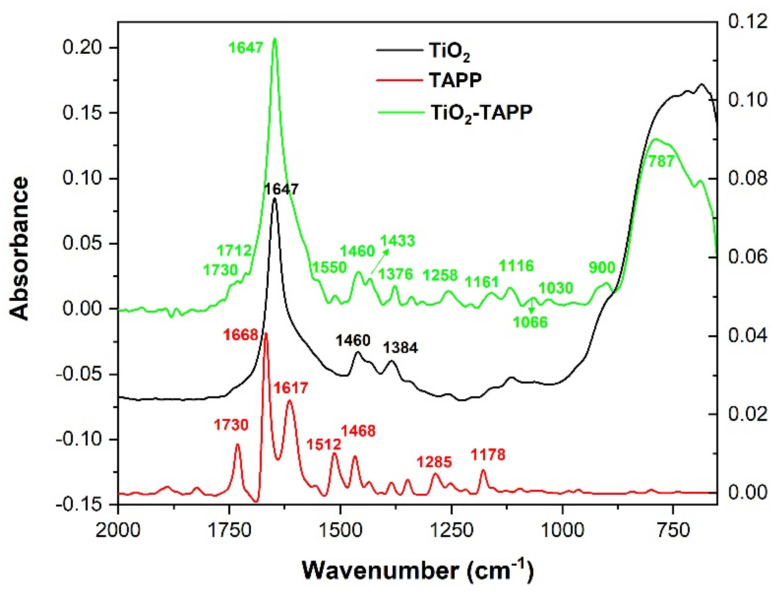
FTIR–ATR spectra of TiO_2_ NPs, TAPP, and TiO_2_ NPs loaded with TAPP. Left axis corresponds to TiO_2_-TAPP spectrum, while right axis corresponds to TiO_2_ and TAPP spectra. TiO_2_ spectrum is vertically translated.

The FTIR-ATR spectrum of TAPP (Figure 4) presents absorption bands that can be assigned based on calculated frequencies to stretching and deformation vibrations of chemical bonds from porphyrin rings, as well as from the aminophenyl radicals, or from both.

The absorption bands that arise at wavenumbers over 3400 cm^−1^ can be assigned to stretching vibrations of NH bonds. The domain 3000–3350 cm^−1^ contains bands that originate in the CH stretching vibrations from the porphyrin ring, as well as from the aminophenyl radicals. NH bending vibrations contribute to the appearance of absorption bands below 1730 cm^−1^ in the FTIR-ATR spectrum of TAPP. In the range 1250–1670 cm^−1^, there is also a contribution of the CC stretching vibrations to the rise of the absorption bands. CN stretching vibrations participate in the appearance of bands with maxima between 960 and 1470 cm^−1^. CC bending vibrations give rise to absorption bands with maxima between 960 and 1120 cm^−1^. CH bending vibrations contribute to the appearance of the absorption bands in the range of 740–1670 cm^−1^.

Furthermore, in Figure 4, the recorded IR spectrum of TAPP is compared with the ones recorded for TiO_2_ NPs and for TiO_2_ NPs loaded with TAPP (TiO_2_-TAPP).

FTIR–ATR spectrum of TiO_2_ NPs presents one absorption band with the maximum at 1647 cm^−1^, followed by one shoulder at 1580 cm^−1^, two lower intensity peaks at 1460 cm^−1^ and 1384 cm^−1^, and one last, also large, band between 850–600 cm^−1^. This last band has its maximum at 686 cm^−1^ and it is preceded by a shoulder at 900 cm^−1^. The large absorption band having the maximum at 3275 cm^−1^ is due to OH stretching vibration along with CH stretching vibrations and overtones of the C=O stretching vibrations. C=O stretching vibrations from the carboxylate groups are responsible for the appearance of the absorption bands with maxima at 1647 cm^−1^ and 1580 cm^−1^. Carboxylate and alkyl groups come from the precursors used in the synthesis of TiO_2_, namely titanium isopropoxide and isopropyl alcohol [47,48]. The absorption bands with maxima at 1460 cm^−1^ and 1384 cm^−1^ are due to CH_2_ bending vibrations. Vibrational modes of Ti–O–Ti, along with CH out-of-plane deformation vibrations from alkyl groups, may be responsible for the appearance of the large absorption band between 650 and 1000 cm^−1^ [47,48].

Figure 4 shows the spectral modifications that are occur during the functionalization of TiO_2_ NPs with TAPP. One important modification is the increase in intensity of the peak at 1647 cm^−1^. To ensure that this increase is not due to a difference in the thickness of the sample, the ratio between the intensities of the TiO_2_-TAPP peak and the TiO_2_ peak at 1647 cm^−1^ was compared with a similar ratio calculated for the intensities at 787 cm^−1^, where the maximum of the absorption band was recorded for TiO_2_-TAPP. For this, the ratio $R_{\tilde{\nu }}$, was determined as follows:

(1) $$R_{\tilde{\nu }}=\frac{A_{\;\mathrm{TiO}2-\mathrm{TAPP}}}{A_{\mathrm{TiO}2}}$$

The ratio calculated for the absorbances recorded at 1647 cm^−1^, $R_{1647}$, was 4.05, while $R_{787}$ was 1.77, demonstrating that the difference in the maxima of absorbance is not caused by a variation in samples thickness. This shows that in the rise of the peak at the 1647 cm^−1^ can contribute, along with C=O stretching vibrations and OH bending vibrations from TiO_2_, CC stretching vibrations, and CH bending vibrations from TAPP.

Prior to the peak at 1647 cm^−1^, two bands with shoulder appearance are observed in the spectrum of TiO_2_-TAPP at 1730 cm^−1^ and 1712 cm^−1^. The absorption band with the maximum at 1730 cm^−1^ is present in the TAPP spectrum and can be attributed to NH_2_ bending vibrations in the aminophenyl radicals, but its intensity is much lower in the TiO_2_-TAPP spectrum, suggesting a disruption of NH bonds in these radicals. The band at 1712 cm^−1^ is distinctive from TAPP and TiO_2_ absorption bands, and can be attributed to the interaction of C=O stretching vibrations of TiO_2_ and C=N stretching vibrations, and CC stretching vibrations of TAPP.

At the same time, the absorption bands of TAPP with maxima 1668 cm^−1^ and 1617 cm^−1^ are no longer present in the IR spectrum of TiO_2_-TAPP, suggesting the disruption of NH bonds in the NH_2_ group from aminophenyl radicals.

The disappearance of the peaks at 1512 cm^−1^ and 1468 cm^−1^ indicates a reduction of CH bending, NH bending, and CC stretching vibrations. This suggests a stiffening of the porphyrin ring, which would appear if TiO_2_ binds with the pyrroles from the TAPP ring. A similar binding between NPs and porphyrin derivatives was also proposed in [39,49].

The shoulder arising at 1550 cm^−1^ in TiO_2_-TAPP IR spectrum is due to NO stretching vibrations [44,45]. The appearance of this type of vibration indicates the formation of chemical bonds between oxygen from TiO_2_ and nitrogen, either from TAPP porphyrin rings or from aminophenyl radicals of TAPP molecules.

The increase of the peak at 1433 cm^−1^ can be attributed to the C=C bond in the porphyrin ring [50], suggesting the presence of the TAPP bound to the TiO_2_ surface, but also to the appearance of NO-stretching vibrations [44,45].

The formation of the TiO_2_-TAPP complex is also demonstrated by the rise of a peak at 1258 cm^−1^. This absorption band appears due to CC and CN stretching, NH bending and CH bending vibrations, but also OH deformation and CO stretching vibration interactions.

The rise of absorption bands in the range 1175–1300 cm^−1^ can be assigned to NO-stretching vibrations [44,45]. This type of chemical bond would appear by adsorbing TAPP on TiO_2_ and bond formation between TiO_2_ and pyrroles from porphyrin ring or TiO_2_ and aminophenyl radicals.

CH bending vibrations, CN stretching, and CC bending vibrations are responsible for the appearance of the absorption bands with maxima at 1161 cm^−1^, 1116 cm^−1^, 1066 cm^−1^, and 1030 cm^−1^ in the IR spectrum of TiO_2_-TAPP.

The absorption bands that appear in the FTIR-ATR spectrum of TiO_2_-TAPP between 650–1000 cm^−1^ are due to association of Ti–O–Ti vibrational modes with NH bending, CH bending, CC bending, and CN stretching vibrations from TAPP. In addition, NO deformation vibrations can contribute to the appearance of absorption bands in the range 840–895 cm^−1^ [44,45]. Zoltan et al. reported [51] a broadening of the band observed in the range 600–1000 cm^−1^ in the IR spectrum of TiO_2_ functionalized with a porphyrin derivative, suggesting N–O–Ti interactions in the material surface.

The modifications observed in the IR spectrum of TiO_2_-TAPP indicate the formation of N–O–Ti bonds, which demonstrate the functionalization of the NPs with TAPP molecules. The IR spectrum demonstrates the disruption of part of the NH_2_ groups indicating that TAPP binds to TiO_2_ through aminophenyl radicals. Simultaneously, the comparison between TAPP and TiO_2_-TAPP spectra reveals a stiffening of the porphyrin ring and a decrease of NH bonds, indicating the formation of N–O–Ti bonds between the pyrroles from porphyrin ring and NPs.

**Figure 5:**
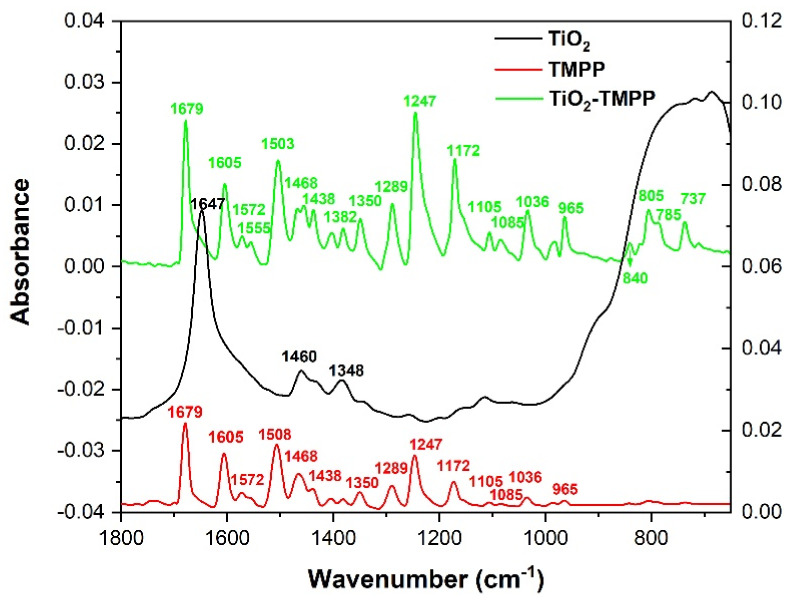
FTIR–ATR spectra of TiO_2_ NPs, TMPP, and TiO_2_ NPs loaded with TMPP. Left axis corresponds to TiO_2_-TMPP spectrum, while right axis corresponds to TiO_2_ and TMPP spectra. The TiO_2_ spectrum is vertically translated.

Table 4 presents the frequencies observed in the FTIR-ATR spectrum of TMPP (Figure 5) compared with the calculated frequencies, as well as the assignation of the vibrational bands to the responsible chemical bonds.

The range of 3000–3320 cm^−1^ presents absorption bands that can be attributed to CH stretching vibrations. CC stretching vibrations, from both porphyrin ring and/or methoxyphenyl radicals, contribute to the appearance of bands between 900–1680 cm^−1^. The bands observed in the region 900–1680 cm^−1^ also have origins in the CH bending vibrations. NH bending vibrations give rise to absorption bands with maxima between 900–1605 cm^−1^. The presence of absorption bands in the 900–1410 cm^−1^ region is also due to CN stretching vibrations from the porphyrin ring or from both porphyrin and methoxyphenyl radicals. CO stretching vibrations contribute to the formation of bands at wavenumbers between 1060–1280 cm^−1^.

In Figure 5, the FTIR-ATR spectrum of TiO_2_-TMPP shows the absorption bands characteristic to TMPP, as well as modified peaks that show the loading of TiO_2_ NPs with TMPP.

The main modifications are represented by the increase in intensity of the peaks between 960 and 1400 cm^−1^, as well as the appearance of new absorption bands with maxima at 840 cm^−1^, 805 cm^−1^, 785 cm^−1^, and 737 cm^−1^.

The possible increase of CC and CN stretching vibrations and CH bending vibrations from the porphyrin ring is responsible for the rise of the absorption band at 1382 cm^−1^. The increase of the peak at 1350 cm^−1^ suggests an increment in CC stretching and CH bending vibrations. Potential increment of CH and NH bending vibrations, as well as CN and CC stretching vibrations, causes the rise of the peak at 1289 cm^−1^. The rise of the absorption band at 1247 cm^−1^ indicates an ascent in CC and CO stretching vibrations, along with CH bending vibrations from methoxyphenyl radicals, as well as CC and CN stretching vibrations and CH and NH bending vibrations from the porphyrin ring. A rising trend of CH bending vibrations, together with CN and CC stretching vibrations, is accountable for the increment of the absorption band at 1172 cm^−1^. Similarly, the increase of CH bending vibrations are responsible for the rise of the peak at 1105 cm^−1^. The rise of the band at 1085 cm^−1^ is due to an increment of CO and CC stretching vibrations along with CH bending vibrations. The increase of CC and CN stretching vibrations from the porphyrin ring, along with CH bending vibrations, are responsible for the upsurge of the absorption bands with maxima at 1036 cm^−1^ and 965 cm^−1^.

The rise of the absorption bands in the region 1150–1360 cm^−1^ may be attributed to the CO stretching vibrations of TMPP in interaction with the OH deformation vibration of possible bonds that can be formed between TMPP and TiO_2_ [44,45]. NO stretching vibrations may also contribute to the appearance of bands in this range, indicating the formation of bonds between the pyrroles in the porphyrin ring and TiO_2_ NPs [39,49].

The new absorption bands that appear in the IR spectrum of TiO_2_-TMPP with maxima at 840 cm^−1^, 805 cm^−1^, 785 cm^−1^, and 737 cm^−1^ are due to the overlay of CC, CN, CH, and NH bending vibrations, as well as NO deformation vibrations, OH deformation, and CO stretching vibration interactions.

The IR spectrum of TiO_2_-TMPP evidences the functionalization of TiO_2_ NPs with TMPP. The appearance of new absorption bands attributed to NO vibrations indicates that TMPP can bind to TiO_2_ via N–O–Ti interactions between the pyrroles of the porphyrin ring and NPs. The increase of the absorption bands assigned to CO vibrations suggest the possibility to form O–C–O–Ti bonds if TMPP binds to TiO_2_ through methoxyphenyl radicals.

**Figure 6:**
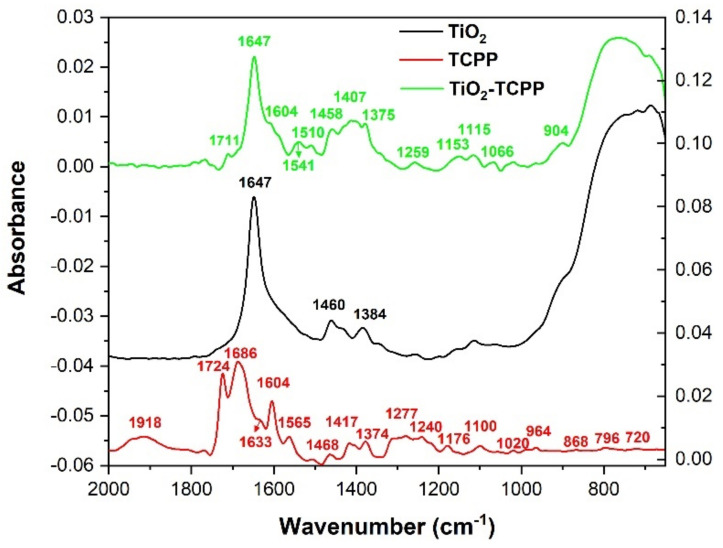
FTIR–ATR spectra of TiO_2_ NPs, TCPP, and TiO_2_ NPs loaded with TCPP. Left axis corresponds to the TiO_2_-TCPP spectrum, while right axis corresponds to TiO_2_ and TCPP spectra. The TiO_2_ spectrum is vertically translated.

The assigned vibrations of TCPP for each observed in the FTIR-ATR spectrum (Figure 6) and calculated frequency are presented in Table 5, evidencing what parts of the molecules the responsible chemical bonds belong to (porphyrin ring, carboxyphenyl radicals, or both).

The absorption peaks between 3070–3320 cm^−1^ are due to stretching vibrations of CH chemical bonds from porphyrin ring or from carboxyphenyl radicals. C=O stretching vibrations contribute to the formation of two absorption bands with maxima at 1724 and 1918 cm^−1^. OH bending vibrations give rise to bands in the areas 1720–1920 cm^−1^ and 670–1370 cm^−1^. CC stretching vibrations influence the appearance of peaks between 960–1730 cm^−1^. CH bending vibrations participate in the formation of absorption bands in the range 670–1690 cm^−1^. The absorption bands observed in the FTIR-ATR spectrum in the range 670–870 cm^−1^ and at 1059 cm^−1^, 1565 cm^−1^, and 1604 cm^−1^ are also due to NH bending vibrations. The stretching vibrations of CN bonds participate in the appearance of peaks observed at the following frequencies: 964, 1374, and 1506 cm^−1^; CN bending vibrations contribute to the formation of the peak at 1403 cm^−1^. The absorption bands observed in the domain 960–1680 cm^−1^ are also due to CC stretching vibrations. The bending vibrations of CC chemical bonds participate in the appearance of peaks between 670–800 cm^−1^ and in the range 1170–1230 cm^−1^. CO bending vibrations, along with other vibrations, are responsible for the formation of absorption bands between 760–800 cm^−1^ and at 1374 cm^−1^. Besides these vibrations, bending vibrations of the OC=O group are responsible for the appearance of the peak observed at 673 cm^−1^ in the FTIR-ATR spectrum of TCPP.

The modifications induced by adsorption of TCPP on TiO_2_ NPs in the IR spectrum, in comparison to the spectra of TCPP and TiO_2_, can be seen in Figure 6.

FTIR-ATR spectrum of TiO_2_-TCPP presents the absorption bands characteristic to TiO_2_ NPs, but also important differences that suggest the loading of TCPP on TiO_2_.

The peak at 1647 cm^−1^, due to C=O stretching vibrations from carboxylate groups remaining from TiO_2_ precursors, is followed by a shoulder with a maximum at 1604 cm^−1^. This band is due to CC ring stretching vibrations along with CH and NH bending vibrations of TCPP.

A superposition of bands appears in the region 1350–1560 cm^−1^, with maxima at 1541 cm^−1^, 1510 cm^−1^, 1458 cm^−1^, 1407 cm^−1^, and 1375 cm^−1^. CC stretching vibrations, CN stretching vibrations from porphyrin ring, as well as CH, NH, and CN bending vibrations characteristics to TCPP, which participate in the formations of these absorption bands.

In the region 650–950 cm^−1^, one large absorption band may be observed, with the maximum at 765 cm^−1^, and another shoulder with the maximum at 901 cm^−1^. These bands may appear due to superposition of Ti–O–Ti vibrational modes with NH, CH, CC, CO bending vibrations of TCPP, as well as CC and CN stretching from TCPP porphyrin ring.

A significant element represents the disappearing of some characteristic bands for TCPP, namely the peaks at 1918 cm^−1^, 1724 cm^−1^, 1686 cm^−1^, and 1633 cm^−1^, as well as the ones between 1210–1315 cm^−1^. These modifications are an indicative of reduction in C=O stretching vibrations and OH bending vibrations, as well as a decrease of the vibrations coming from carboxyphenyl radicals, suggesting that TCPP binds to TiO_2_ through these radicals. Similar findings suggest that TCPP is chemisorbed on the surface of TiO_2_, forming Ti–O–C=O bonds [52,53].

The changes observed in the FTIR-ATR spectrum of TiO_2_-TCPP suggest that the loading of TiO_2_ NPs with TCPP may be similar to the one presented in [54], where carboxyl-functionalized porphyrins create surface-anchored connecting carboxylates with TiO_2_ NPs through the carboxylic acid radical.

Modifications observed in the FTIR-ATR spectra of the complexes formed by TiO_2_ loading with porphyrin derivatives TAPP, TMPP, and TCPP, respectively, suggested that the adsorption of porphyrin may involve the pyrroles in the porphyrin ring, as suggested in [39,49], or may implicate the radicals of the porphyrin derivative [54].

### 2.4. Singlet Oxygen Generation

Based on the analysis of the absorption spectra, the concentration of the porphyrin photosensitizer in the nanoparticle suspensions was established to determine their quantum efficiency for the generation of singlet oxygen, a reactive species used in PDT.

The singlet oxygen quantum yield was determined using a standard solution of protoporphyrin IX (PPIX) in DMF as a reference. The time-resolved phosphorescence signal of the generated singlet oxygen (λ = 1270 nm) was recorded under the same conditions for porphyrin solutions, suspensions with porphyrin-functionalized TiO_2_ nanoparticles, and the PPIX standard in DMF. The quantum yield was calculated according to the formula:

(2) $$\Phi =\Phi_{ref}\frac{I}{I_{ref}}\frac{A_{ref}}{A}\frac{n^{2}}{n_{ref}^{2}}\frac{\tau_{ref}}{\tau }$$

where *Φ* is the singlet oxygen generation yield, *I* is the singlet oxygen phosphorescence intensity, *A* is the sample absorption, *τ* is the singlet oxygen lifetime, *n* is the refractive index of the solvent, and the ref index corresponds to the reference sample values. The phosphorescence intensity is obtained by extrapolating the mono-exponential phosphorescence kinetics fitting curve at *t* = 0.

The quantum yield for singlet oxygen generation for PPIX in DMF was determined as 0.85 by measuring its phosphorescence kinetics relative to PPIX in ethanol, which has *Φ* = 0.92 [55].

**Figure 7:**
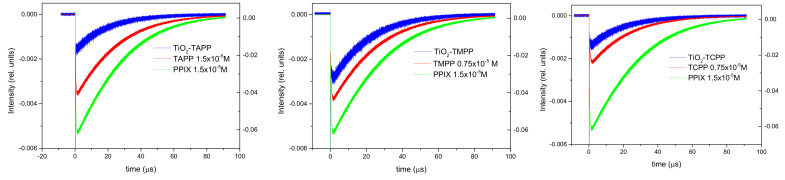
Time-resolved phosphorescence signals for single oxygen generated by TAPP, TMPP, TCPP, and their conjugates with TiO_2_ in comparison with PPIX used as reference.

Figure 7a–c exhibits the time-resolved phosphorescence signals for singlet oxygen generated by porphyrin solutions and suspensions in DMF containing TiO_2_ NPs complexes with TAPP, TMPP, and TCPP, in comparison with PPIX, which was used as standard. TiO_2_ suspensions did not present a phosphorescence signal and generation of singlet oxygen when excited with 532 nm radiation.

Following the processing and analysis of phosphorescence signals, by using Equation (2), the quantum yield for singlet oxygen generation was obtained for the studied porphyrins and the suspensions of their complexes with TiO_2_. The photophysical data on singlet oxygen generation were summarized below in Table 6.

It can be noticed that the quantum yield for generation of singlet oxygen by the studied porphyrins is smaller compared with their conjugates with TiO_2_ for TAPP and TMPP, while for the TCPP-TiO_2_ NPs complex, a decrease is observed.

A potentation effect on photodynamic activity can take place by shifting the response of TiO_2_ in the visible range [56].

The TiO_2_ nanoparticles can be photosensitized and produce ROS by electron transfer from the visible light-excited PS. While photogenerated electrons react with dissolved oxygen molecules to produce •O_2_^−^, photogenerated holes mostly react with the water in the surroundings to produce OH radicals. Some •O_2_^−^ can form ^1^O_2_ by reacting with the holes [57]. From these two species, ROS produced by TiO_2_ is ^1^O_2_ rather than•O_2_^−^ [58].

In contrast, the interaction of PS with TiO_2_ and the energy transfer is related to the radicals as anchoring groups of the porphyrins [54,59]. The carboxyl groups favorized these interactions in the adsorption of TCPP on TiO_2_ surface [59], which was how it was observed from the FTIR spectra as well. These stronger interactions compared with the methyl or amino porphyrin derivatives possibly favorized the formation of OH rather than ^1^O_2_ radicals. Further investigations should be made to evaluate the contribution of each species in the ROS formed.

The best value of quantum yield was obtained for TiO_2_-TMPP; still, all of the complexes of the studied porphyrins with TiO_2_ NPs exhibited a good efficiency in singlet oxygen generation. Due to TiO_2_ non-toxicity to living organisms, its stability in water, its photocatalytic activity [36], and its potential as a drug carrier, the studied complexes are good candidates for further photodynamic therapy in vitro assays either on tumoral cell lines or microorganisms such bacteria.

## 3. Materials and Methods

**Figure 8:**
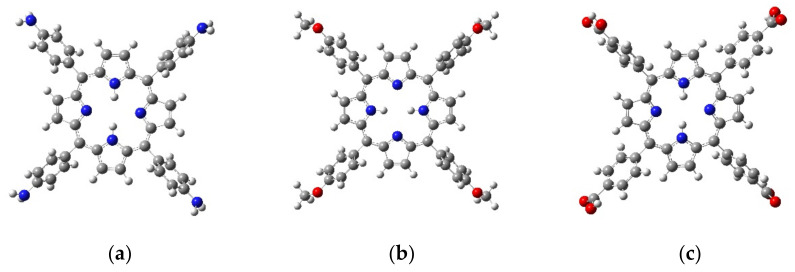
The chemical structure of the studied porphyrins: (**a**) TAPP, (**b**) TMPP, (**c**) TCPP (blue—N atoms, red—O atoms, grey—C atoms, white—H atoms).

The following porphyrin derivatives, which have good potential for single oxygen generation, have been selected for obtaining the nanocomplexes: 5,10,15,20–(Tetra-4-aminophenyl) porphyrin (TAPP), 5,10,15,20–(Tetra-4-methoxyphenyl) porphyrin (TMPP), and 5,10,15,20- (Tetra-4-carboxyphenyl) porphyrin (TCPP); in addition, protoporphyrin IX (PPIX) was used as the standard. The chemical structures of the studied porphyrins are depicted in Figure 8.

TAPP, TMPP, and TCPP were supplied by PorphyChem SAS, Dijon, France; PPIX was supplied by Sigma Aldrich (Darmstadt, Germany); TiO_2_ NPs as anatase, with 99.995% purity and 17 nm diameter, were provided by Nanografi (Ankara, Turkey).

Solutions of the four porphyrins at concentrations between 0.75 × 10^−5^ M–1.5 × 10^−5^ M were prepared in dimethylformamide (DMF). Solvent DMF was purchased from Merck (Darmstadt, Germany).

The functionalization of TiO_2_ NPs with the selected porphyrins was performed according to the methods detailed in the literature [32,50,60]. Mixtures of TiO_2_ and porphyrins were prepared using a 1:3 ratio of 0.1 mg/mL suspensions of TiO_2_ in DMF with 2.5 × 10^−5^ M porphyrin solutions in DMF. The resulting mixtures were sonicated for 5 h and were then kept at room temperature in the dark for 24 h. In the next step, the suspensions were centrifuged for 10 min at 12,000 rpm, and the supernatant was removed in order to eliminate the number of porphyrins that were not adsorbed on the nanoparticles’ surfaces. The solid deposit was then redispersed in the same volume of DMF using the ultrasound bath for 30 min.

The morphology and structural characteristics of the synthesized TiO_2_ and TiO_2_-porphyrin samples were investigated by scanning electron microscopy (SEM) using an Apreo S microscope from FEI (Thermo Fisher Scientific, Waltham, MA, USA) with an energy-dispersive X-ray spectroscopy (EDS) system, fixed silicon detector, and integrated Peltier element as a cooling system. SEM analyses were performed at an acceleration voltage of 15 kV, in the magnification range of 100,000×, at 10 cm working distance, and a spot beam of 3. For EDS, the used beam spot was 6.5–7, the working distance was 10 cm, and the dead time was 30 s. It was also operated at 10 kV acceleration voltage and 6.3 pA electrical current.

The hydrodynamic size and stability of the nanoparticle suspensions, as well as the porphyrin-functionalized TiO_2_ NPs suspensions, were analyzed by dynamic light scattering (DLS) using Nanoparticle Analyzer SZ-100V2 (Horiba, Kyoto, Japan). This employs a diode-pumped solid-state laser emitting at 532 nm and a scattering angle of 173°. Hydrodynamic size measurements were performed in triplicate for 1 mL volume of each suspension placed in a quartz cuvette cell. For zeta potential measurements, an electrophoretic cell with carbon electrodes (6 mm) was used with a lower volume of solution.

The porphyrin loading for the functionalized nanoparticles was assessed by absorption spectroscopy. The absorption spectra of porphyrin solutions and their suspensions in quartz cells of 1 cm pathlength were measured in the 300–700 nm spectral range using a spectrophotometer Lambda 950 (Perkin-Elmer, Waltham, MA, USA) with 1 nm spectral resolution.

FTIR-ATR spectroscopy measurements have also been conducted to highlight the emergence of nanocompound complexes with porphyrin derivatives, and to assess the possible modification of their functional groups. The FTIR-ATR spectra of the three porphyrin derivatives, as well as spectra of their complexes with TiO_2_ NPs, were recorded. For this purpose, a volume varying between 100 and 200 μL from each sample was dried on polyethylene (PE) FTIR-cards. Individual 5 µL drops of the sample were dried repeatedly in order to obtain a greater thickness of the sample, which was an implicitly higher optical path. IR spectra were registered using the ATR module of the FTIR NicoletTM iSTM50 spectrometer (Thermo Fisher Scientific, Waltham, MA, USA) in the spectral range 4000–650 cm-1, with 4 cm-1 spectral resolution. Each spectrum represents a mediation of 16 recordings. The sample was placed facing the ZnSe crystal (internal reflection at an incidence angle of 42°, diameter 1.5 mm, refractive index 2.4, and penetration depth 2.03 µm at 1000 cm^−1^).

The theoretical IR spectra were calculated using Gaussian09 software [43]. The molecular structure was subjected to geometry optimization followed by the calculation of vibrational wavenumbers using the density functional theory (DFT). The hybrid functional B3LYP method with 6-311G(d,p) basis set was used.

The attainment of quantum yield for singlet oxygen generation was performed using the experimental system described elsewhere [61] and based on the measurement of the time-resolved phosphorescence of singlet oxygen at 1275 nm. The excitation source was the second harmonic generation of the pulsed Nd:YAG laser (Minilite II, Continuum), which was emitted at 532 nm with 3 mJ excitation energy and 10 Hz frequency. The phosphorescence was measured using an electronically cooled NIR photomultiplier (Hamamatsu H-10330) whose signal was digitized by an oscilloscope (Tektronix DPO 7254). The sample was placed in a photometric cuvette with an optical pathlength of 1 cm. The phosphorescence was collected at a right angle and was filtered to eliminate wavelengths other than 1270 nm by a suitable optical arrangement (aperture lenses and interference filters) placed in the front of the photomultiplier.

## 4. Conclusions

The spectral investigations of TiO_2_ nanoparticle complexes with TAPP, TMPP, and TCPP porphyrins was performed. The morphological characterization of the samples revealed the formation of porphyrin-TiO_2_ complexes.

The UV-Vis absorption spectra of the complexes showed the characteristic bands of porphyrins and these were used to determine the concentration of adsorbed porphyrins on TiO_2_ NPs surface.

FTIR-ATR spectra of each porphyrin–TiO_2_ complex showed vibrational bands characteristic to TiO_2_ NPs together with peaks typical for porphyrins, but also included changes that suggested the loading of TiO_2_ with porphyrins. These analyses indicated that the functionalization of NPs with porphyrin derivatives TAPP, TMPP, and TCPP, respectively, involves either the pyrroles in the porphyrin ring, or it implies the bonding of TiO_2_ with the radicals of the porphyrin derivative.

In the case of TiO_2_-TAPP, the appearance of absorption bands assigned to NO vibrations suggested that TAPP binds to TiO_2_ via N–O–Ti bonds. The binding could take place either through aminophenyl radicals—considering that the IR spectrum shows a decrease in the signal originating from NH_2_ groups—or through the pyrroles of the porphyrin ring because a rigidity of the porphyrin ring and a reduction of NH bonds was also observed. Evidence of N–O–Ti bonds also appeared in the IR spectrum of TiO_2_-TMPP, suggesting that TMPP can bind to TiO_2_ through the pyrroles of the porphyrin ring. The rise in the absorption bands assigned to CO vibrations indicated the possibility of forming O-C-O-Ti bonds between the methoxyphenyl radicals and NPs. IR spectrum of TiO_2_-TCPP suggested that the binding of TCPP to TiO_2_ takes place via carboxyphenyl radicals, forming Ti–O–C=O bonds.

The studied porphyrin complexes with TiO_2_ NPs showed good efficiency in singlet oxygen generation. Further investigations will be devoted to the evaluation of the contribution of other reactive oxygen species generated by complexes due to the presence of the TiO_2_ photocatalyst.

The complexes analyzed in this study represent promising candidates for further photodynamic therapy in vitro assays either on tumoral cell lines or microorganisms such as bacteria.

## Figures and Tables

**Table 1:** Dimension of the particles and EDS analysis of the TiO_2_ and TiO_2_-porphyrins samples.

Sample	Mean Size/nm SEM	Element	Weight %	Atomic %
TiO_2_	20	C K	5.02	6.48
		O K	28.01	46.37
		Ti K	66.97	47.15
TiO_2_-TAPP	43	C K	12.31	21.62
		N K	2.68	4.03
		O K	42.06	55.26
		Ti K	42.95	19.09
TiO_2_-TCPP	21	C K	3.19	7.54
		N K	1.63	3.31
		O K	27.73	49.17
		Ti K	67.45	39.98
TiO_2_-TMPP	31	C K	3.34	8.02
		N K	2.14	4.40
		O K	29.49	43.59
		Ti K	65.03	43.99

**Table 2:** The mean hydrodynamic size and zeta potential of the samples.

Sample	Mean Size (nm)	Standard Deviation (nm)	Polydispersity Index	Zeta Potential (mV)
TiO_2_	559.1	126.9	0.403	−50.3
TiO_2_-TAPP	1213.7	233.0	0.733	−46.8
TiO_2_-TMPP	1148.4	322.5	0.954	−44.1
TiO_2_-TCPP	1225.8	274.1	0.956	−44.0

**Table 3:** Experimental and calculated frequencies for TAPP.

Observed Frequency (cm^−1^)	Calculated Frequency (cm^−1^)	Assigned Vibrations
741	702	NH_2_ bending and CH bending from aminophenyl radicals
801	749	NH bending; CH bending
842	858	NH bending; CH bending
964	986	CC bending; CN stretching; CH bending
1095	1017	CH bending; CN stretching; CC bending
1126	1158	NH_2_ bending; CH bending
1158	1183	CN stretching; CH bending
1178	1208	CH bending; NH bending
1196	1209	CH bending
1216	1217	NH bending; CH bending
1252	1272	CN stretching; NH bending; CH bending; CC stretching
1285	1301	CN stretching; NH bending; CH bending
1348	1372	CH bending; NH bending; CC stretching; CN stretching
1386	1388	CH bending; NH bending; CC stretching; CN stretching
1437	1428	CH bending; CC stretching
1468	1499	CC and CN stretching from porphyrin ring; CH bending
1500	1548	CH bending; NH bending; CC stretching
1512	1554	CH bending; CC stretching
1555	1594	CC stretching; CH bending; NH bending; CN stretching
1617	1656	CC stretching; NH_2_ bending; CH bending
1668	1657	CC stretching; NH_2_ bending; CH bending
1715	1691	NH_2_ bending
1730	1692	NH_2_ bending
3029	3131	CH stretching
3118	3158	CH stretching from aminophenyl radicals
3213	3160	CH stretching from aminophenyl radicals
3337	3237	CH stretching from porphyrin ring
3421	3519	NH_2_ symmetrical stretching
3440	3543	NH stretching from porphyrin ring

**Table 4:** Experimental and calculated frequencies for TMPP.

Observed Frequency (cm^−1^)	Calculated Frequency (cm^−1^)	Assigned Vibrations
965	986	CC and CN stretching from porphyrin ring; CH bending
1036	1003	CC and CN stretching from porphyrin ring; CH bending
1085	1064	CO stretching; CC stretching; CH bending
1105	1130	CH bending
1172	1196	CH bending; CN stretching; CC stretching
1247	1273	CC and CO stretching; CH bending from methoxyphenyl radicals; CC and CN stretching, CH and NH bending from porphyrin ring
1289	1277	CH bending; NH bending; CN stretching; CC stretching
1350	1323, 1331	CC stretching; CH bending
1382	1374	CC stretching, CN stretching, CH bending from porphyrin ring
1403	1392	NH bending; CH bending; C=C and CN stretching from porphyrin ring
1438	1478	CH_3_ wagging; CH bending
1468	1503, 1505	CC stretching; CH_3_ bending
1508	1540	CH bending; CC stretching
1605	1596	CC stretching from porphyrin ring; CH bending; NH bending
1679	1650	CC in-ring stretching from methoxyphenyl radicals; CH bending
3002	3000	CH_3_ symmetrical stretching
3032	3057	CH_3_ asymmetrical stretching
3069	3132	CH stretching from CH_3_
3105	3174	CH stretching from methoxyphenyl radicals
3118	3204	CH stretching from methoxyphenyl radicals
3320	3259	CH stretching from porphyrin ring
-	3548	NH stretching

**Table 5:** Experimental and calculated frequencies for TCPP.

Observed Frequency (cm^−1^)	Calculated Frequency (cm^−1^)	Assigned Vibrations
673	658	NH, CH, OH, OC=O and CC bending
720	760	CH, NH and CC bending
765	780	CC, CO, OH, CH and NH bending
781	787	CC, CO, OH, CH and NH bending
796	789	CC, CO, OH, CH and NH bending
868	827	CH and NH bending
964	986	CC and CN stretching from porphyrin ring; CH bending
1020	1096	CC stretching; CH and OH bending from carboxyphenyl radicals
1059	1107	CC stretching; CH bending; NH bending
1100	1191	CH bending; OH bending
1176	1215	CC bending; CH bending; OH bending
1222	1303	CC bending; CH bending; OH bending
1240	1312	CC stretching; CH bending; OH bending
1277	1318	CC stretching; CH bending; OH bending
1314	1336	CH bending; OH bending
1374	1376	CC, CO, OH and CH bending from carboxyphenyl radicals; CC and CN stretching, and CH bending from porphyrin ring
1403	1427	CC stretching; CN bending; CH bending
1417	1430	CC stretching from carboxyphenyl radicals; CH bending
1468	1431	CC stretching from carboxyphenyl radicals; CH bending
1506	1508	CC stretching; CH bending; CN stretching from porphyrin ring
1565	1595	CC stretching; CH bending; NH bending
1604	1601	CC ring stretching; CH bending; NH bending
1633	1646	CC stretching; CH bending from carboxyphenyl radicals
1686	1648	CC stretching; CH bending from carboxyphenyl radicals
1724	1803	C=O stretching; OH bending; CC stretching
1918	1834	C=O stretching; OH bending
3079	3154	CH stretching from carboxyphenyl radicals
3123	3180	CH stretching from carboxyphenyl radicals
3275	3187	CH stretching from carboxyphenyl radicals
3319	3260	CH stretching from porphyrin ring
-	3547	NH stretching

**Table 6:** The photophysical data on singlet oxygen generation.

Compound/Solvent DMF	^1^O_2_ Lifetime (µs)	^1^O_2_ Yield
TAPP	24	0.54
TCPP	21.2	0.74
TMPP	24	0.92
TiO_2_-TAPP	19.5	0.65
TiO_2_-TCPP	19.5	0.62
TiO_2_-TMPP	20.53	0.96

## Data Availability

Not applicable.
